# Ligand assisted reprecipitation of formamidinium–guanidinium lead iodide 2D perovskite nanowires

**DOI:** 10.1039/d5nr04638f

**Published:** 2025-11-27

**Authors:** Liam Van Gaal, Shuichi Toyouchi, Mayank Goyal, Nadine Schrenker, Sumea Klokic, Peiran Wang, Heinz Amenitsch, Emmanuel Lhuillier, Sara Bals, Bapi Pradhan, Elke Debroye

**Affiliations:** a Department of Chemistry, KU Leuven Celestijnenlaan 200F 3001 Heverlee Belgium bapi.pradhan@kuleuven.be elke.debroye@kuleuven.be; b Sorbonne Université, Faculté des Sciences, CNRS, Institut des Nano-Sciences de Paris (INSP) 4 pl Jussieu 75005 Paris France; c Electron Microscopy for Materials Science (EMAT) and NANOlab Center of Excellence, University of Antwerp 2020 Antwerp Belgium; d Institute of Inorganic Chemistry, Graz University of Technology Stremayrgasse 9/IV Graz 8010 Austria

## Abstract

Two-dimensional (2D) lead halide perovskites have emerged as a promising alternative to their three-dimensional counterparts, offering superior ambient stability and enhanced moisture resistance. Additionally, A-site multi-cation perovskites have gained attention for their ability to improve stability and enhance optoelectronic device performance. Despite these advantages, the synthesis of multi-cation 2D perovskites has traditionally been limited by complex and time-intensive methods, hindering their broader application potential. In this work, we demonstrate the use of a ligand-assisted reprecipitation synthesis approach to produce high-quality 2D formamidinium–guanidinium lead iodide perovskites. By varying the ratio of surface capping ligands, aspect-ratio-tuned nanowires (NWs) were obtained. Phase-pure NWs were confirmed from grazing-incidence wide-angle X-ray scattering and 4D scanning transmission electron microscopy. A single particle optical study pointed out that these confined structures of 2D perovskites were shown to exhibit non-linear optical (NLO) anisotropy in the form of third-harmonic generation and two-photon photoluminescence along the growth direction of the NWs. To demonstrate practical applicability, flexible photodetectors based on these NWs were fabricated, exhibiting a two-order-of-magnitude increase of conductance under UV illumination (405 nm) upon increasing the irradiance from 1 mW cm^−2^ to 1 W cm^−2^, with sub-50 µs response times. Power-dependent photoconductivity measurements further revealed that photo-carrier generation is limited by a bimolecular recombination process originating from band-to-band recombination, highlighting the intrinsic charge transport dynamics of the system.

## Abstract 2

Formamidinium–guanidinium lead iodide nanowires synthesized *via* ligand-assisted reprecipitation, demonstrating flexible photodetection and enhanced nonlinear optical response.

## Graphical abstract

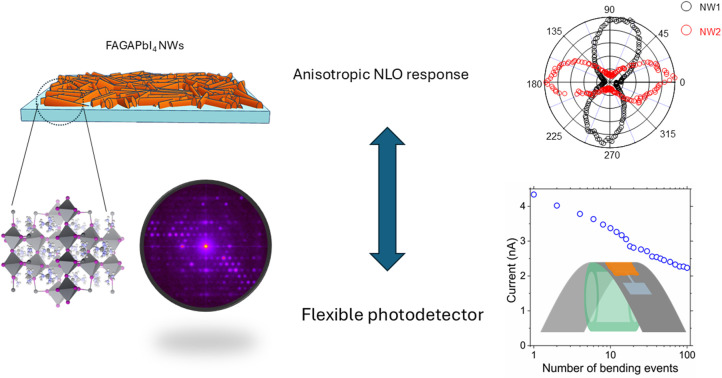

## Introduction

Three-dimensional (3D) APbX_3_ lead halide perovskites (LHPs), where A is a monovalent cation and X is a halide, are at the center of current materials research owing to their immense potential in light-harvesting and light generation, with remarkable progress realized within a short period of time.^1,2^ Low-cost precursors and facile, scalable solution-process fabrication techniques lead to the fabrication of semiconductors with superior photophysical properties fueling the LHPs optoelectronic research.^3–5^ Much focus is now being spent on fabricating perovskite optoelectronic devices combining high-efficiency with long-term operational durability.^3,6–8^ However, the imminent structural and chemical instability of 3D LHPs under moisture, oxygen, heat and light is a major fundamental issue limiting practical device applications.^9,10^ Hence, novel perovskite structures need to be synthesized combining good structural and optical ambient stability with excellent optoelectronic properties. To this end, dimensionality engineering is being investigated to enhance the stability of LHPs.^11^ In particular, 2D perovskites, where hydrophobic organic spacers form insulating layers between lead halide octahedra, offer significantly improved environmental stability.^12,13^ These insulating layers not only protect the inorganic framework from external stressors like moisture and oxygen but also mitigate structural defects, contributing to the overall robustness of the material.^14^ Concomitantly, the morphology of LHPs can be controlled to further acquire the desired properties. So far, nanocrystalline 2D perovskites have been reported in the form of nanocubes,^15^ quantum dots,^16^ nanosheets,^17^ and nanorods.^18^ Overall, research on 2D perovskite nanowires (NWs) remains limited, likely due to the inherent challenges in achieving precise control over their one-dimensional growth while applying facile synthesis protocols.^19^

The main benefit of NWs arises from their unique structure leading to anisotropic optoelectronic properties.^20^ These characteristics have enabled their use in diverse applications where direction-dependent charge transport, light–matter interactions, and quantum confinement play a crucial role.^21–26^ Although nanowire architectures are commonly associated with 3D perovskite phases, our work shows that NWs can also be fabricated from 2D perovskite structures, combining dimensional confinement with the anisotropic forms of both structure and morphology. 2D perovskites are highly likely to exhibit third-order nonlinear optical (NLO) processes due to their unique excitonic landscape. On the one hand, the excitons are delocalized and display Wannier-like behaviour in the inorganic slabs.^27,28^ On the other hand, in the perpendicular direction, excitons are localized displaying Frenkel-like behaviour.^28^ The resulting high oscillator strengths promote the generation of high exciton densities. These large populations, in turn, facilitate strong exciton-exciton interactions causing them to deviate from ideal bosons, leading to strong third-order optical nonlinearities.^28,29^ NLO properties play a key role in various applications such as ultrashort laser pulse generation through processes like mode-locking,^30,31^ facilitate frequency conversion,^32,33^ and enhance bioimaging by enabling deeper tissue penetration and improved contrast.^34^ While substantial progress has been made in enhancing the linear optical properties of halide perovskites, research into their nonlinear optical characteristics remains in its infancy.^35,36^

The large surface area of perovskite NWs significantly boosts their light-harvesting capabilities, while the spatial confinement of charge carriers within the highly crystalline 1D morphology further improves charge separation, transport, and extraction.^37–39^ Altogether, the above-mentioned advantages of NWs make them ideal candidates as photoactive materials for photodetector (PD) applications.^39–41^ Furthermore, NWs are known to exhibit improved mechanical properties giving rise to opportunities for the fabrication of flexible PD devices.^40^

To date, multiple methods have been explored for the fabrication of NW perovskite materials, prime examples are the hot injection method, solvothermal synthesis and vapor growth.^39,42^ Most of these methods require high temperatures, controlled atmosphere, and dedicated setups. Consequently, they are energy-inefficient and not suitable for large-scale production. To this end, we optimized the synthesis protocol of the facile room-temperature ligand-assisted reprecipitation (LARP) method,^43–45^ to achieve 2D FAGAPbI_4_ perovskite NWs.^46^ Incorporating two A-site cations into a pure 2D perovskite, however, introduces inherent structural complexity. While single-cation 2D perovskites adopt a straightforward A_2_PbI_4_ structure, dual-cation systems such as FAGAPbI_4_ are prone to phase segregation into individual perovskite phases. In particular, FA can form 3D FAPbI_3_,^47^ while GA may crystallize into 3D GAPbI_3_ and 2D GA_2_PbI_4_.^48,49^ Achieving phase-pure FAGAPbI_4_ nanoparticles therefore requires precise control over reactivity and surface ligand binding.

The crystal structure of these FAGAPbI_4_ NWs is composed of corrugated Pb–I layers and GA and FA cations situated in the interlayer space.^50^ Building on the inherent stability benefits of 2D perovskites (as discussed above), GA incorporation offers an additional route to lattice reinforcement. Together with FA, GA cations are strong hydrogen-bond donors *via* their ammonium group, leading to a higher relative content of hydrogen bonds strengthening the crystal lattice.^50,51^ Overall, the relatively small size, nearly zero dipole character, and strong coordination make GA less insulating than the long-chain organic spacers typically used in 2D perovskites, thereby reducing the dielectric contrast and enabling improved electronic coupling between adjacent inorganic layers.^52,53^ In this work, the use of FAGAPbI_4_ as active NLO material is further explored. Due to its wire-like morphology, a unique anisotropic response with a polarization ratio approaching unity is observed for both two-photon photoluminescence (TPPL) and third harmonic generation (THG). Furthermore, the use of our material in a flexible photodetector device is demonstrated, proving the potential optoelectronic application of 2D perovskite NWs. Power-dependent measurements and temperature-dependent analysis were conducted to further study the devices.

## Results and discussion

The synthesis of aspect-ratio tuned NWs requires specific control over growth kinetics and precise regulation with high temperature reactions.^54,55^ Herein, we have used a room temperature (RT) ligand assisted reprecipitation (LARP) technique to synthesise aspect-ratio tuned NWs with a uniform morphology of 2D perovskites. LARP is a scalable method in which a precursor solution is mixed with an antisolvent to induce reprecipitation, while added ligands regulate particle size, shape, and stability.^45,56–58^ Stoichiometric molar precursor salts formamidinium iodide (FAI), guanidinium iodide (GAI), and lead iodide (PbI_2_) were dissolved in *N*,*N*-dimethylformamide, in presence of capping ligands (octanoic acid and octylamine), resulting in a transparent yellow precursor solution following stirring at RT. A very small amount of this precursor (∼15 µL) was swiftly added to an excess of toluene (10 mL) under vigorous stirring (further details regarding the precursor composition and synthesis procedure are provided in the SI). Toluene has been used as antisolvent which induces rapid crystallization of the perovskite at RT.^59^ The apparent colour of the reaction mixture gradually transforms from yellow to orange and finally red with prolonged stirring. After ∼10 minutes of stirring, the colour of the solution does not change further, implying the completion of the reaction (Fig. S1). Systematic variation in the ligand ratio and precursor concentration was found to tune the length and width of the NWs (Fig. S2), aligning with the observed morphology in single crystals.^50^ It should be noted that slight changes in precursor concentration or ligand ratio significantly affect the aspect ratio and phase purity of the obtained nanowires. This was already apparent from their optical appearance; while samples such as FGPI-4,20 were deep red, indicative of the targeted FAGAPbI_4_ phase, others like FGPI-4,0 were yellow to light orange, suggesting the formation of competing phases. Accordingly, not all resultant NWs showed the successful formation of the phase-pure FAGAPbI_4_ structure (*vide infra*). Following the similar wire-like structure for all samples, and considering the relatively intense red colour of the FGPI NWs synthesized with 4/20 ratio of OctAm/OctAc v/v ratio, we have selected this sample for further study. Reactions without the use of capping ligand produced uncontrolled crystallinity.

**Fig. 1:**
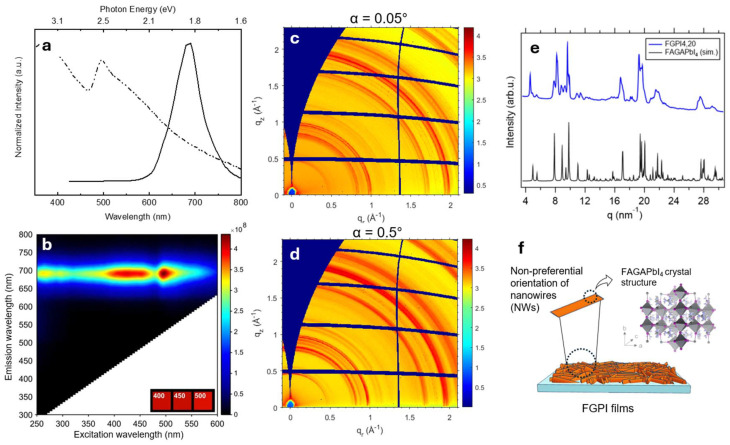
Optical and structural characterization of the FGPI-4,20 sample. (a) UV-Vis absorption (dotted line) and photoluminescence (solid line, excitation wavelength: 400 nm) measurements of the FGPI-4,20. (b) PL emission map of FGPI-4,20. GIWAXS patterns of FGPI-4,20 measured at grazing incidence angles of *α* = 0.05° and 0.5° are shown in panels (c) and (d), respectively. (e) Azimuthal integration of the GIWAXS pattern at *α* = 0.5°, shown alongside the simulated pattern for single-crystal FAGAPbI_4_.^50^ (f) Schematic representation of crystalline NWs deposited on the substrate, illustrating their non-preferential orientation. The crystal structure corresponds to FAGAPbI_4_.^50^

The UV-Vis absorption and PL spectra of the FGPI NWs (Fig. 1a) displays an emission peak at 688 nm and the UV-Vis spectrum displays a sharp excitonic peak as expected for pure 2D perovskites due to their strong dielectric and quantum confinement.^60^ FGPI-4,20 has a Full Width at Half Maxima (FWHM) of 0.19 eV. Similar broad emission features have previously been reported for single-crystal FAGAPbI_4_ and were attributed to self-trapped exciton (STE)-related recombination.^50^ STE-based emission has also been reported for other corrugated 2D perovskite structures, further supporting this interpretation.^61,62^ The observed emission maximum clearly differs from that of 3D FAPbI_3_ nanorods, which typically shows a more narrow red-shifted PL.^63–65^ While a minor contribution from 3D FAPbI_3_ present as isolated nanocrystals or domains cannot be entirely excluded, hence the overall spectral characteristics support a predominantly 2D corrugated nature of the FGPI nanowires. In the PL emission map of FGPI-4,20 (Fig. 1b) two pronounced intensity maxima are observed at different excitation wavelengths both coinciding with the absorption maxima in the UV-Vis spectrum. The excitation–emission map (Fig. 1b) reveals fluctuating intensity for the same broad PL band (with an invariant position centered at ∼688 nm) mirroring the UV-Vis absorption spectrum.

To assess the crystalline properties of the nanowires after deposition onto substrates, grazing-incidence wide-angle X-ray scattering (GIWAXS) measurements were performed on the FGPI-4,20 NWs. The incident angle (*α*) was varied between 0.05° and 0.5° to selectively probe the near-surface region and the underlying bulk material, respectively, and to evaluate whether the nanowires exhibit any preferential orientation relative to the substrate surface, an effect commonly encountered for perovskites.^66,67^ The GIWAXS patterns at *α* = 0.05° and 0.5° display isotropic intensity distributions (Fig. 1c and d), indicating the absence of preferential alignment and suggesting that the nanowires are randomly oriented across the substrate surface. To better resolve the structural characteristics of the bulk and reduce surface scattering and peak broadening effects, azimuthal integration was carried out on GIWAXS data acquired at *α* = 0.5°. As shown in Fig. 1e, the experimental pattern is in good agreement with the simulated diffraction pattern which is based on single-crystal data for FAGAPbI_4_ reported by Nazarenko *et al.*,^50^ confirming phase purity of the deposited NWs. However, the presence of weak, unassigned reflections at *q* = 6.47 nm^−1^, 8.23 nm^−1^ and 11.4 nm^−1^ suggests the possible existence of a minute amount of a FAPbI_3_-related side phase, potentially forming a mixed 2D/3D structure. The GIWAXS data confirm that the deposited nanowires consist primarily of the 2D FAGAPbI_4_ phase, with only weak reflections suggesting the presence of minor additional Pb–I-based phases, such as FAPbI_3_, GA_2_PbI_4_, or GA_2_PbI_5_ (Fig. S3).^68^ Though this cannot be definitively confirmed, the GIWAXS analysis overall reveals a highly isotropic orientation of the nanowire crystallites. A schematic depiction of this structural arrangement is provided in Fig. 1f. Furthermore, the FGPI-4,0 NWs show structural heterogeneity. The GIWAXS data for both FGPI NWs are overlapped and compared with different crystallographic phases for both samples (Fig. S4 and S5). It is clearly visible that FGPI-4,0 also exhibits mixed-phase characteristics: the FAGAPbI_4_ phase is a good match, with lower contribution from GA_3_PbI_5_. The reflections associated with FAPbI_3_ (at 9.87, 19.75, and 22.09 nm^−1^) are strong in FGPI-4,20, particularly for regions near the surface (*α* = 0.05° and 0.1°), but are significantly less pronounced in FGPI-4,0 (Fig. S4). This suggests reduced FAPbI_3_ formation in FGPI-4,0. Additionally, several weak reflections observed in FGPI-4,0 do not correspond to any of the known FAGAPbI_4_, GA_3_PbI_5_, or GAPbI_3_ phases, (Fig. S5), presumably due to insufficient ligand content, which limits the stabilization of isolated nanocrystals (as observed for the FPGI-4,20), leading instead to a more structurally heterogeneous mixture.

To elucidate the elemental composition and chemical environment of the FGPI NWs, XPS measurements were performed (Fig. S6). All spectra were calibrated to the C 1s peak at 284.8 eV (adventitious aliphatic carbon).^69^ The C 1s envelope was deconvoluted into three components: the main aliphatic carbon at 284.8 eV, a feature at 286.1 eV consistent with C–N contributions likely originating from FA^+^/GA^+^ cations^70,71^ and possibly also surface amine-containing ligands, and a high-energy component at 288.8 eV which can be ascribed to carbonyl/carboxylate species (O–C=O) from surface ligands^71,72^ (octanoic acid). The N 1s region was best fit with two peaks at 398.4 and 400.2 eV. The higher-binding component (400.2 eV) dominates and is assigned to protonated nitrogen species (FA^+^ and GA^+^),^73,74^ while the lower-binding signal (398.4 eV) is consistent with neutral amine moieties at the nanowire surface^75,76^ (*e.g.* octyl amine). The O 1s envelope contains two components at 531.9 and 533.0 eV; these are typically assigned to O=C and O=C–O respectively,^75,77^ which could be due to the octanoic ligands present at the surface, however it should be noted that these peaks could also arise in air exposed samples.^77^ Together, the C, N and O signatures indicate coexistence of protonated A-site cations and carboxylate/amine ligands interacting with the surface. In the Pb 4f region, the sharp doublet is characteristic of Pb^2+^ in the perovskite lattice, with very weak shoulders attributable to trace metallic Pb^0^.^71,78^ The I 3d spectrum shows the expected doublet positions for iodide anions, confirming an intact Pb–I framework.^71^ Overall, the XPS data verify the presence of FA and GA cations, amino-carboxylate surface ligands and a predominantly PbI_6_^4−^ perovskite lattice, with evidence for ligand-Pb coordination and minor Pb reduction at the surface.

Furthermore, Raman spectroscopy was employed to gain further insight into the structural characteristics of the FGPI nanowires (Fig. S7). A strong and broad peak around 113 cm^−1^ is typically assigned to δ-FAPbI_3_, and its absence in our spectra rules out a prominent contribution from this phase.^79^ Instead, we observe several distinct peaks in the low-frequency region, which is consistent with reports on other 2D perovskite structures. In layered A_2_PbI_4_-type materials, this spectral window is associated with lattice vibrations of the Pb–I octahedra.^80,81^ Such features usually manifest as multiple resolved peaks in contrast to the single dominant δ-FAPbI_3_ mode. Further theoretical calculations and mode analysis will be required to unambiguously assign the individual Raman bands in FGPI which is beyond the scope of the present study.

**Fig. 2:**
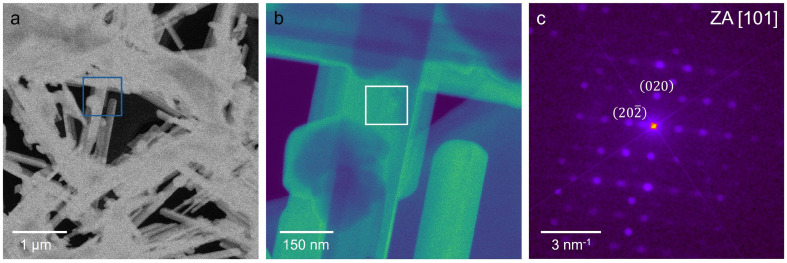
STEM imaging of FGPI-4,20 NWs. (a) HAADF overview image of the FGPI sample revealing the nanowire morphology. (b) Virtual ADF image reconstructed from a nano-beam 4D STEM dataset. The image depicts a close-up of the area indicated with the blue rectangle in panel a. (c) Nano-beam 4D STEM diffraction pattern. The diffraction patters were summed up from the 4D STEM dataset over the region indicated in the white rectangle in panel b. The diffraction pattern matches the [101] zone axis of FAGAPbI_4_.^50^

Furthermore, we investigated the FGPI-4,20 NWs *via* Scanning Transmission Electron Microscopy (STEM) and nano-beam 4D STEM (Fig. 2). Perovskite materials with organic cations are prone to degradation due to electron beam irradiation.^82^ Therefore, in addition to conventional high-angle annular dark-field (HAADF) STEM imaging, we also employed low-dose nano-beam 4D STEM using a direct electron detector. This enables us to obtain a local nano-beam electron diffraction pattern using a low electron dose (<10 e^−^ Å^−2^). The HAADF image in Fig. 2a reveals the nanowire morphology. The diameter of the NWs ranges from 50–200 nm. From the 4D STEM data-set a virtual annular dark-field (ADF) image was reconstructed, which is depicted in Fig. 2b. The nano-beam 4D STEM diffraction pattern (Fig. 2c) agrees with the [101] zone axis of the FAGAPbI_4_ crystal structure that was reported for single crystals by Nazarenko *et al.*^50^

In contrast, the FGPI-4,0 sample revealed a less uniform nanowire morphology (Fig. S8). Microprobe 4D STEM measurements indicated that even though a nanowire morphology was obtained, they exhibited diffraction patterns that matched either the perovskite FAPbI_3_ or its δ-phase. Furthermore, the detection of an additional unidentified phase (Fig. S9) confirms the structural heterogeneity as observed in the GIWAXS measurements (Fig. S5). This structural heterogeneity explains the yellow-to-light orange appearance of FGPI-4,0 and highlights the sensitivity of phase formation to precursor concentration and ligand ratio. These results confirm that only samples synthesized under optimized conditions yield phase-pure FAGAPbI_4_ nanowires, whereas small deviations readily promote competing iodide phases.

**Fig. 3:**
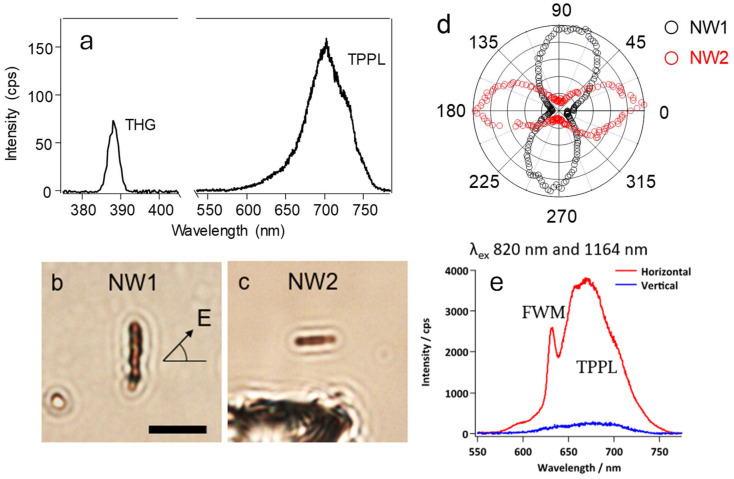
Nonlinear spectroscopy of FGPI NWs: excitation polarization dependence on two-photon photoluminescence (TPPL), third harmonic generation (THG), and four-wave mixing (FWM). (a) Nonlinear spectra of FGPI NWs. TPPL peak around 690 nm and THG peak around 388 nm were observed under fs laser irradiation (1164 nm, 200 fs, 0.2 GW cm^−2^ for TPPL and 0.84 GW cm^−2^ for THG, respectively). (b and c) Optical transmission images of FGPI NWs, that are vertically (b. NW1) and horizontally aligned (c. NW2). Scale bar is 2 μm. (d) TPPL intensities polar plots of NW1 (black circles) and NW2 (red circles) under fs laser irradiation (820 nm, 120 fs, 0.35 GW cm^−2^). (e) Emission spectra of NWs with different orientations. The broad peak at 690 nm is due to TPPL, the sharp peak at 632 nm can be attributed to FWM.

So far, 3D MHPs have demonstrated great potential for not only light emission but also NLO applications. NLO properties are critical for advanced photonic applications^83^ such as frequency conversion,^32,33^ optical switching,^83^ and signal processing,^84^ enabling faster and more efficient communication technologies. However, the nonlinearity of 2D perovskites nanoparticles has not been investigated well at the single particle level. NLO properties of the FGPI NWs were explored in the present study on a single NW (FGPI-4,20). Fig. 3a shows nonlinear spectra of FGPI NWs obtained under 1164 nm femtosecond laser irradiation (details of the NLO response experiments are provided in the SI). A TPPL peak around 690 nm and a THG peak around 388 nm were clearly observed. These NLO signals were further examined by excitation power dependence (Fig. S10, SI). The excitation power ranged from 12.4 MW cm^−2^ to 6.8 GW cm^−2^. The corresponding excitation power dependence on TPPL and THG intensities show a power law of 1.85 (Fig. S10c, SI) and 3.08 (Fig. S10d, SI), respectively, corroborating our assignments. These NLO signals were observed with relatively weak excitation power compared to the previously reported 3D and 2D perovskites,^83,85^ where input powers on the order of several to several tens of GW cm^−2^ were typically required, indicating that FGPI NWs possess relatively high third-order NLO susceptibility, *χ*^(3)^. This can be further explained by the enhanced exciton binding energies and oscillator strength in quantum well (2D) structures, which result in significantly increased third-order nonlinear optical susceptibilities (*χ*^(3)^).^86–88^ The strong Coulomb interactions in these 2D structures lead to higher exciton binding energies, enhancing the nonlinear optical response by increasing the efficiency of exciton-exciton interactions.^89^ Additionally, the high oscillator strength in these systems reflects efficient light–matter coupling, further boosting the third-order NLO susceptibility.^90^

The NLO signals of the FGPI NWs exhibit a significant optical anisotropy. Fig. 3b and c shows the optical transmission images of FGPI NW1 and NW2 used in the polarization dependence experiment, which are vertically and horizontally aligned, respectively. TPPL signals under 820 nm fs laser irradiation were measured with various angles of laser polarization. The polarization was rotated by using a half-waveplate mounted in a motorized holder (see details in SI). The polarization dependence on PL intensity confirmed that the TPPL intensity of NW1 with vertically polarized light (*E* ∼90 degree, parallel to the NW long axis, *I*_*∥*_) is more than 300 times higher than with horizontally polarized light (*E* ∼0 degree, perpendicular to the NW long axis, *I*_⊥_), suggesting the strong NLO anisotropy of the NW1 (Fig. 3d, black circles). The TPPL polarization ratio, *ρ* = (*I*_*∥*_ − *I*_⊥_)/(*I*_*∥*_ + *I*_⊥_) is calculated to be 0.99. Similar optical anisotropy has been reported by other researchers on one-photon excitation one-photon PL (OPPL).^91,92^ However, in the case of OPPL, the polarization ratio is at around 0.8.^14,93,94^ This high polarization ratio, indicative of strong NLO anisotropy, has only been reported in a handful of other studies on perovskites.^95–98^ The polarization ratio observed in TPPL seems to be extraordinarily high compared with previous studies.^96^ This strong anisotropy presumably arises from the inherent properties of the 2D perovskite together with the 1D morphology of the nanowires.^99^ To further verify the NLO anisotropy reproducibility, the same experiment was done for a horizontally aligned NW2 (Fig. 3c). NW2 also showed strong NLO anisotropy on TPPL (Fig. 3d, red circles, *ρ* = 0.94), while the maximum TPPL intensity was observed with horizontal polarization (*E* ∼0 degree). As expected, for aggregated NWs, no NLO anisotropy was observed (Fig. S11a and b). Additionally, a strong anisotropic NLO response was noted when observing the four-wave mixing (FWM) of the NWs (Fig. S12). Here, the NWs were irradiated with both a 820 nm and a 1164 nm laser, which were spatiotemporally overlapped. Next to the peak at 690 nm due to TPPL, a sharp peak at 632 nm was found due to FWM (Fig. S12c). The intensity of the emitted NLO light was strongly dependent on the orientation of the NWs. Furthermore, THG under 1164 nm fs laser irradiation also exhibits strong NLO anisotropy as shown in Fig. S11c and d. The polarization ratio is calculated to be 0.99. These experimental data demonstrated that the nonlinearity of the TPPL, THG, and FWM processes emphasizes the substantial optical anisotropy with an improved polarization ratio. Hence, our study demonstrated that FGPI NWs represent a good architecture for the development of polarization-sensitive (nonlinear) optoelectronic devices.

**Fig. 4:**
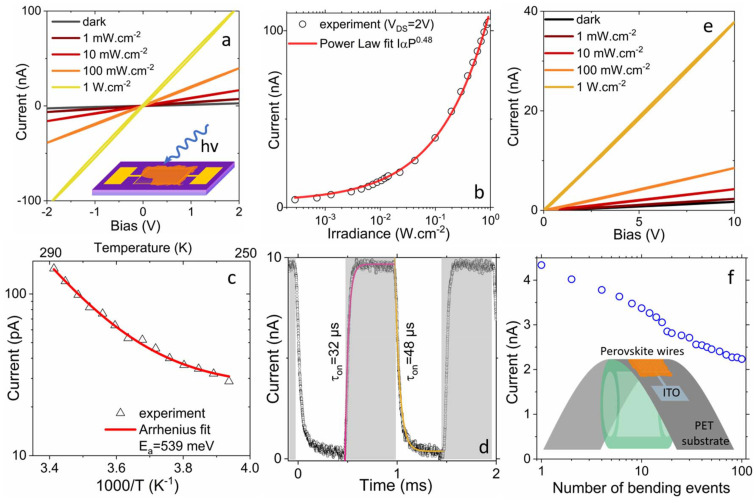
Photodetection of FGPI NWs. (a) *I*–*V* curves in the dark and under illumination under various incident power for a thin film of perovskite NWs deposited on Si/SiO_2_/Au electrodes. Illumination is ensured by a 405 nm laser diode. The inset is a scheme of the device. (b) Current as a function of the irradiance of the blue laser power for a thin film of the perovskite NWs deposited on Si/SiO_2_/Au electrodes. (c) Current as a function of the temperature for a thin film of perovskite NWs deposited on Si/SiO_2_/Au electrodes. An Arrhenius fit of the data leads to an activation energy of 540 meV. (d) Current as a function of the time while the incident light blue illumination is turned on and off for a thin film of perovskite NWs deposited on Si/SiO_2_/Au electrodes. The part in grey corresponds to the laser on. (e) *I*–*V* curves under dark condition and under illumination under various incident power for a thin film of perovskite NWs deposited on PET/ITO electrodes. Illumination is ensured by a 405 nm laser diode. (f) Current under 20 V applied bias for a thin film of perovskites NWs deposited on PET/ITO electrodes as a function of the number of bending events experienced by the film. The inset is a scheme of the bent device. Measurements are conducted after re-flattening.

The solution processability of these NWs offers ease of optoelectronic device fabrication. We have tested the transport and phototransport properties of the perovskite NW array. To do so, we dropcasted a hexane dispersion of these NWs (FGPI-4,20) onto metallic (Au) interdigitated electrodes (25 pairs of digits with a length of 2.5 mm and spacing of 10 µm) deposited onto a Si/SiO_2_ wafer (Fig. 4a, inset). We observe optimal performance of the film without performing any non-solvent washing steps, as such procedures would destroy the sample. This is likely due to the use of a very low concentration of surface ligands compared to conventional colloidal synthesis, where any additional ligand removal step needs to be carefully done to avoid agglomeration. The material appears to be conductive and photoconductive (Fig. 4a). Upon illumination by a 405 nm (≈3 eV) source, far above the material's absorption band gap, we observe a clear increase of the conductance. The latter can reach two orders of magnitude for an irradiance of 1 W cm^−2^. The power dependence of the current with the incident light can be well fitted by a power law, of which the exponent is close to ½ (Fig. 4b). Similar exponent values have been measured for cesium^100^ and formamidinium^101^ based perovskite nanocrystal arrays. Such a half-value for the power dependence of the current unveils that the amount of photo-generated carriers is limited by a bimolecular process (*i.e.* band to band recombination) rather than by trapping (monomolecular process^102,103^).

The temperature dependence on the photocurrent is given in Fig. 4c. The activation energy around room temperature can be obtained from an Arrhenius fit. We estimate its value to be ≈540 meV. Such value has to be compared to half the photoluminescence band gap 1.65 eV. This clearly reveals that the Fermi level lies deep in the material band gap and that only very weak intrinsic doping should be expected in this material. To further confirm the stability and reliability of the device, we performed a photoswitching experiment. We have probed the response time of the material, with the turn-on and -off time to be around 32 µs and 48 µs, respectively, and is actually limited by the chopping blade (Fig. 4d).

Last, we have tested the integration of these solution processable NWs into flexible electronic devices. In this case, the NWs are drop casted onto prepatterned (*i.e.* also interdigitated but only 1 mm long and spaced by 50 µm) tin doped indium oxide (ITO) deposited on a flexible polyethylene terephthalate (PET) substrate. Conductive and photoconductive properties of the film are preserved in spite of the change of electrode work function (Fig. 4e). Upon multiple bending steps, we observe a continuous decay of the array conductance (Fig. 4f). The device performance appears to be mostly limited by the adhesion of the nanowire array onto the electrodes. Further development by synthesizing stable colloidal dispersion of NWs and nanorods *via* additional stronger surface binding ligands and at a higher reaction temperature might overcome these limitations.

## Conclusions

In summary, we have successfully demonstrated the facile room-temperature LARP synthesis of aspect-ratio-tuned nanostructures, more specifically NWs of corrugated 2D perovskite. By controlling the surface ligands, we achieved phase-pure NWs. The phase purity of the NWs was confirmed through GIWAXS and 4D STEM analyses. Our findings at the single particle level reveal that these nanostructures exhibit strong NLO responses, more specifically, THG and TPPL. Additionally, the NLO signal displayed a strong NLO anisotropy. Furthermore, the FGPI NWs were used to fabricate a flexible 405 nm light photodetector, demonstrating a two-order-of-magnitude increase in conductance under an irradiance of 1 W cm^−2^. Power-dependent analysis suggested that photo-carrier generation is driven by a bimolecular process, while temperature-dependent measurements fitted with the Arrhenius model yielded an estimated activation energy of 540 meV. The response times were measured at 32 µs for turn-on and 48 µs for turn-off, highlighting the material's fast photoresponse. Further exploration of these NWs to achieve aligned assemblies will be critical for enhancing polarization-dependent optoelectronic properties in future, bringing us closer to realizing the full potential of 2D perovskite nanostructures in advanced optoelectronic applications.

## Conflicts of interest

There are no conflicts to declare.

## Supplementary Material

NR-017-D5NR04638F-s001

## Data Availability

The data supporting this article have been included as part of the supplementary information (SI). Supplementary information is available. The supplementary information contains: schematic illustration of the LARP synthesis protocol; SEM images of FAGAPbI_4_ nanowires prepared with varying precursor concentrations and ligand ratios; comparison of GIWAXS patterns at different grazing incidence angles alongside simulated single-crystal patterns and phase indexing; XPS and Raman spectra of FGPI nanowires; HAADF-STEM and nano-beam 4D STEM diffraction pattern of the nanowires; TPPL and THG spectra under femtosecond laser excitation with corresponding power dependence; polarization-dependent nonlinear optical responses of individual and aggregated nanowires; orientation-dependent emission spectra highlighting TPPL and four-wave mixing contributions. See DOI: https://doi.org/10.1039/d5nr04638f.
