# Supplementary material for: Ligand assisted reprecipitation of formamidinium–guanidinium lead iodide 2D perovskite nanowires
Source: Nanoscale. 2025 Nov 27;17(48):28123–33. doi: 10.1039/d5nr04638f (PMC12658878; doi:10.1039/d5nr04638f)
Supplement: NR-017-D5NR04638F-s001 [file NR-017-D5NR04638F-s001.pdf]

Supplementary Information (SI) for Nanoscale.
This journal is © The Royal Society of Chemistry 2025

Ligand Assisted Reprecipitation of Formamidinium-Guanidinium Lead Iodide 2D Perovskite Nanowires

Liam Van Gaal[†], Shuichi Toyouchi[†], Mayank Goyal[‡], Nadine Schrenker[⊥], Sumea Klokic[π], Peiran Wang[†], Heinz Amenitsch[π], Emmanuel Lhuillier[‡], Sara Bals[⊥], Bapi Pradhan[†], and Elke Debroye[†]**

[†]Department of Chemistry, KU Leuven, Celestijnenlaan 200F, 3001 Heverlee, Belgium

[‡]Sorbonne Université, Faculté des Sciences, CNRS, Institut des Nano-Sciences de Paris (INSP), 4 pl Jussieu, 75005 Paris, France

[⊥]Electron Microscopy for Materials Science (EMAT) and NANOlab Center of Excellence, University of Antwerp, 2020 Antwerp, Belgium

[π]Institute of Inorganic Chemistry, Graz University of Technology, Stremayrgasse 9/IV, Graz 8010, Austria

Email: *bapi.pradhan@kuleuven.be elke.debroye@kuleuven.be*

Chemicals

Lead iodide (PbI_2, 98%), octylamine (OctAm, 98%), octanoic acid (OctAc, 98%), N,N dimethylformamide (DMF, 99%), toluene (anhydrous, 99.8%) has been purchased from Sigma Aldrich. Formamidinium iodide (FAI) and guanidinium iodide (GAI) has been purchased from Great Solar cell, Australia. The stock solution of FAI and GAI has been stored in the glove box. A small amount of the precursor has stored in N_2 filed desiccator for use. All the chemicals have been used without further purification. All the reaction has been performed at room temperature under ambient condition unless otherwise stated.

Synthesis of FGPI NWs

1 mmol of PbI_2, 1 mmol of FAI, 1 mmol of GAI has been taken in a 20 ml glass vial followed by addition of 10 mL DMF. The mixture is stirred and sonicated till dissolution. 1 mL of these solution is transferred to other vials followed by the addition of different amounts of OctAm and OctAc. The amount of the OctAm(μL)/OctAc(μL) ratio has been varied with the following ratios: 0/0; 0/4; 2/0; 4/0; 4/4; 4/10; 4/20; 4/30, followed by sonication of the precursor solutions. 15 μL of each solution is added to 10 mL toluene under stirring at 1200 rpm for 10 min. Then the resultant nanocrystals have been precipitated by centrifuging at 5000 rpm for 5 min followed by dispersion in toluene. The nanocrystals have been centrifuged again to remove excess surface ligands followed by dispersion in a small volume of toluene and stored under ambient condition in a glass vial for further use.

Characterizations

Synchrotron-based grazing incident wide angle X-ray scattering (GIWAXS)

GIWAXS measurements were performed at the Austrian SAXS beamline at ELETTRA synchrotron in Trieste (Italy) at a photon energy of 8 keV.[1] The beam size was set to 0.1 x 1 mm (V × H) at a sample to detector (Pilatus3 1M, Dectris) distance of 217.3 mm using silver behenate as a reference pattern with the patterns corrected for the fluctuations of the primary intensity. The measurements were performed in air at incident angles of 0.05° and 0.5° to provide depth-sensitive information with the X-ray beam penetrating into the film. The GIWAXS patterns were reconstructed and indexed using the GIXSGUI toolbox.[2] The pattern shown in Figure 1 a-d are represented by orthogonal q_z and q_r reciprocal axes, where q_z denotes the wave vector transfer component normal to the surface and q_r being the total in-plane wave vector transfer. In such a representation a missing wedge appears, since the intensity distribution on an area detector measures the projection of the reciprocal lattice being intercepted by the Ewald sphere onto the detector plane. Azimuthal integration of the GIWAXS pattern to one-dimensional pattern was performed by SAXSDog.[3] Simulation of the single-crystal patterns was done by Mercury, while intensity profile comparison was performed by IGOR Pro (Wavemetrics).

UV-Vis Diffuse Reflectance Spectroscopy absorption measurements of the nanocrystals have been carried out in using a PerkinElmer Lambda 950 UV spectrophotometer with an integrating sphere. The data were recorded at room temperature between 350 and 800 nm with a 1 nm step.

Emission Spectroscopy Photoluminescence (PL) spectra are taken employing the Edinburgh FLS980 equipped with a He-Cd laser used at an excitation wavelength of 420nm.

Fourier-Transform Infrared Spectroscopy (FTIR) spectra are recorded wat the Agilent Technologies FTIR spectrometer. The raman spectra were obtained via a confocal Raman microscope (MonoVista CRS+). An excitation wavelength of 523 nm (8.54 mW laser power) was chosen for all the samples and was directed on the samples through a 50X objective with a numerical aperture of 0.3.

X-ray Photoelectron Spectroscopy (XPS) measurements were obtained using a K-alpha Thermo spectrometer with an Al Kα radiation at 1486.68 eV.

Scanning Transmission Electron Microscopy (STEM) analysis is performed at a Tecnai G2 at 200kV. Nano-beam 4D STEM experiments were conducted using an Amsterdam Scientific Instruments CheeTah T3 (see ref.[4] for more details on the 4D STEM setup).

Scanning Electron Microscopy (SEM) Morphology and composition were further analyzed using an FEI Quanta FEG-250 environmental scanning electron microscope operating at 20 kV, equipped with an energy-dispersive spectrometer. Before measurement, the NW suspension was drop-cast onto a silicon chip.

Nonlinear optics (NLO) Nonlinear optical properties of the NWs have been measured in an inverted optical microscope (Ti-U, Nikon) equipped with a piezoelectric stage (P517.3CL, Physik Instrument). For these measurements, two laser wavelengths were employed at 820 nm and 1164 nm (Inspire HF 100, Spectra-Physics). A femtosecond (fs) laser beam (Spectra-Physics®, Mai Tai HP; 820 nm, 120 fs, 80 MHz) was split into two paths using a 90:10 beamsplitter. The weaker portion was directed into a microscope to irradiate the NWs directly. The stronger portion was sent into an optical parametric oscillator (OPO) (Spectra-Physics®, Inspire HF 100) to generate a tuneable wavelength output. The OPO output (1164 nm, 200 fs, 80 MHz) was then also guided into the microscope. Temporal overlap between the two fs pulses was finely adjusted using an optical delay line. For both experiments, the excitation beams were focused on the samples using an objective lens (60x PlanApo, air, NA 0.95, Nikon). The backscattered NLO signals were corrected by the same objective, and spectra were recorded using a charge-coupled device (CCD) camera (DU920P, Andor) at an operating temperature of −85°C equipped with a spectrograph (iHR320, Horiba). A shortpass filter (ET800SP-2P, Chroma or FES0700, Thorlabs) and a pinhole (100 μm diameter) were positioned in front of the entrance to the spectrograph to cut off the excitation laser.[1]

Photoresponse of the NWs have been carried out on an Al electrode of 200 um spacing and mm in length. The samples have been drop casted into the channel of electrode followed by drying at room temperature to evaporate off all the solvents. The device is connected in a probe station.

Electrodes on Si/SiO_2 wafer: The surface of a Si/SiO_2 wafer (400 nm as thickness) is cleaned by sonication in acetone. The wafer is rinsed with isopropanol and finally cleaned using an O_2 plasma. AZ 5214E resist is spin-coated and baked at 110°C for 90 s. The substrate is exposed under UV though a pattern mask for 2 s. The film is further baked at 125°C for 2 min to invert the resist. Then a 40 s flood exposure is performed. The resist is developed using a bath of AZ 326 for 32 s, before being rinsed in pure water. We then deposit a 3 nm chromium layer and a 40 nm gold layer using a thermal evaporator. The lift-off is performed by dipping the film for

in acetone for 1h. The electrodes are finally rinsed using isopropanol and dried by an air flow. The electrodes are 2.5 mm long and spaced by 10 µm.

Electrodes based on ITO on PET: Indium tin oxide (ITO) coated on polyethylene terephthalate (PET) (≈ 80 nm coating with a 60 Ω/cm² resistance) sheet are purchased from Sigma-Aldrich. The film is rinsed using acetone and then isopropanol before being dried. AZ 5214E resist is spin-coated and then baked for 90 s on a 110°C hot-plate. The film is then exposed to UV for 4 s though a shadow mask. The resist is then developed for 45 s in AZ 726 and rinsed in pure water. The naked ITO is then etched using 25% HCl solution for 15 s and then quickly rinsed in pure water. The lift-off of the resist is made by dipping the substrate in acetone for 5 min and then rinsing the film with isopropanol. The designed electrodes are interdigitated electrodes with 50 µm spacing. Each electrode is itself 50 µm large and 1 mm long. The total active area is 1 mm^2. A side gate electrode is also present for electrolyte gating.

Thin film preparation: The electrodes are rinsed with acetone and isopropanol, and dried using N_2 gun. 4 µL of material is dropcasted on the electrodes and let air dry.

Electrical Measurements: Measurement are conducted in air using a probe station. The sample is connected to a Keithley 2634b source meter, which set the applied bias and measure the current. For illumination, we use a 405 nm blue laser diode from coherent. The power of the diode is tuned either by tuning the applied current or by adding an optical density (OD 2) or by combining the two effects.

For time resolved photocurrent, the sample is set on the cold finger of a cryostat, the laser diode is chopped using a mechanical chopper. The sample is biased using a femto DLPCA 200 amplifier which also magnified the output current from the sample. The signal is finally acquired onto a Tektronix MDO3102 oscilloscope.

For the experiment on flexible substrate, the stability as a function of the number of bending events is tested while the sample is bent with an 8.5±1 mm bending radius.

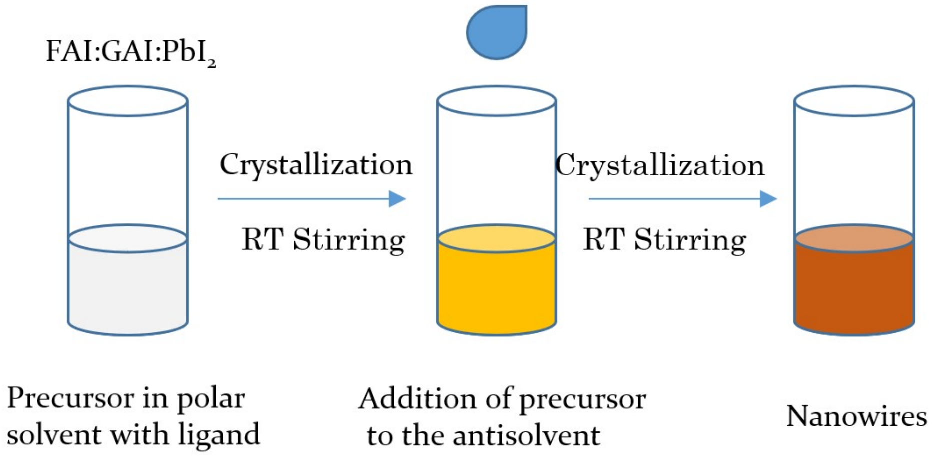

Figure S1. Schematic depiction of the room-temperature LARP synthesis protocol.

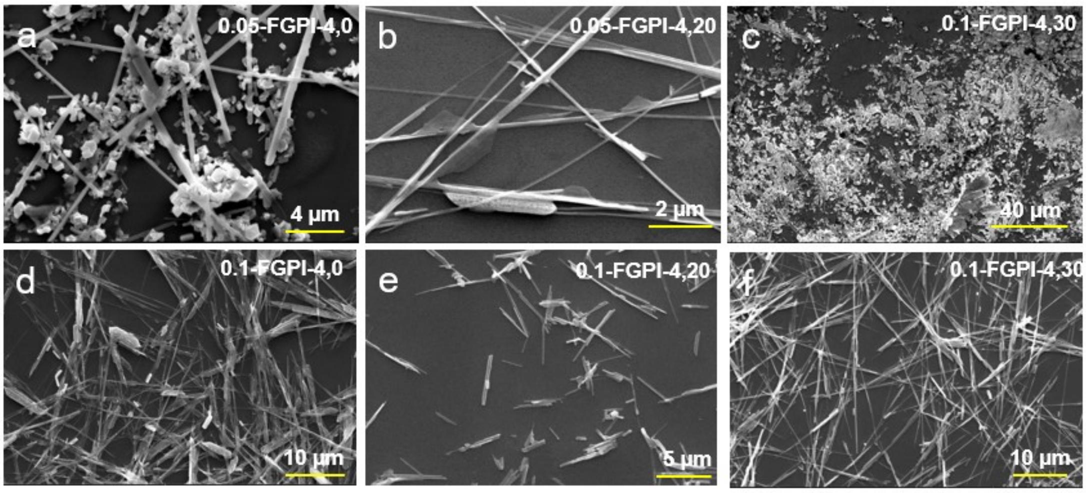

Figure S2. SEM images of the $FAGAPbI_4$ synthesized with different precursor concentrations and ligand ratios. Each image is labelled as a-FGPI-x,y where a denotes the precursor concentration, and x,y the amount of ligand (in μL) as OctAm, OctAc respectively.

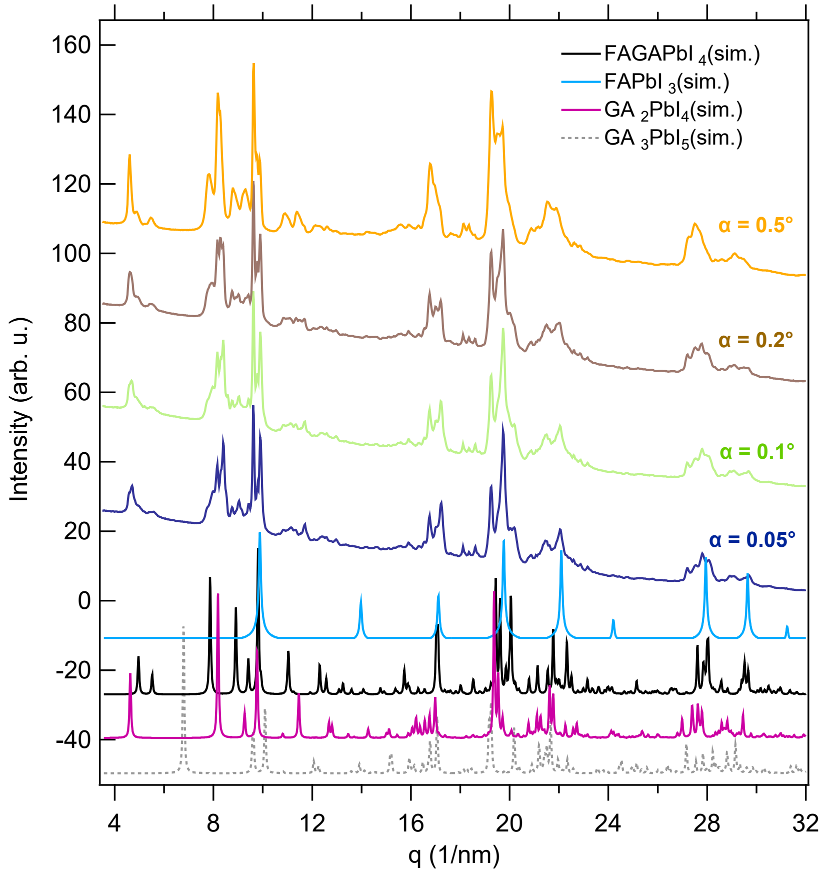

Figure S3. Comparison of GIWAXS patterns of FGPI-4,20 measured at grazing angles of α = 0.05°, 0.1°, 0.2° and 0.5° and simulated pattern for single-crystal $FAGAPbI_4$,$FAPbI_3$, GA_2PbI_4 or GA_2PbI_5.

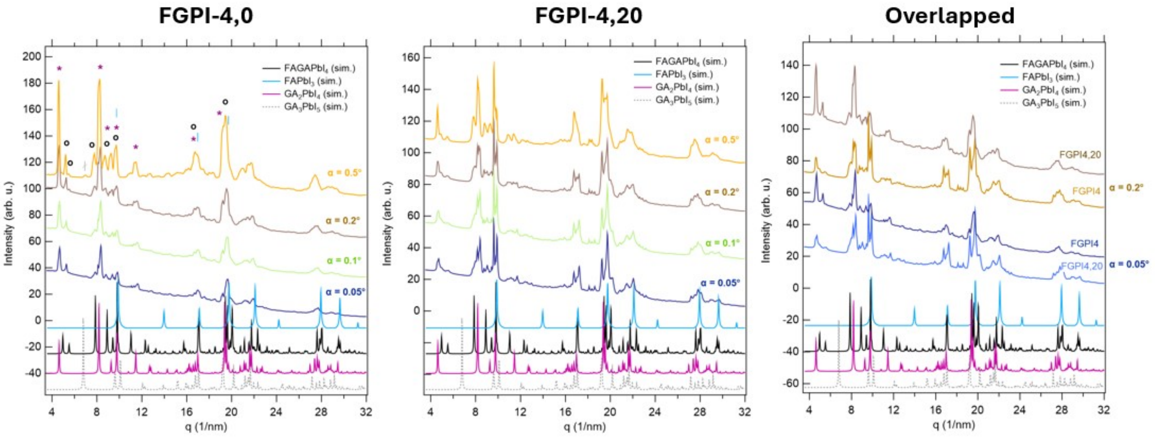

Fig. S4: Phase indexing of FGPI-4,0 and FGPI-4,20 NWs samples with different crystallographic phases.

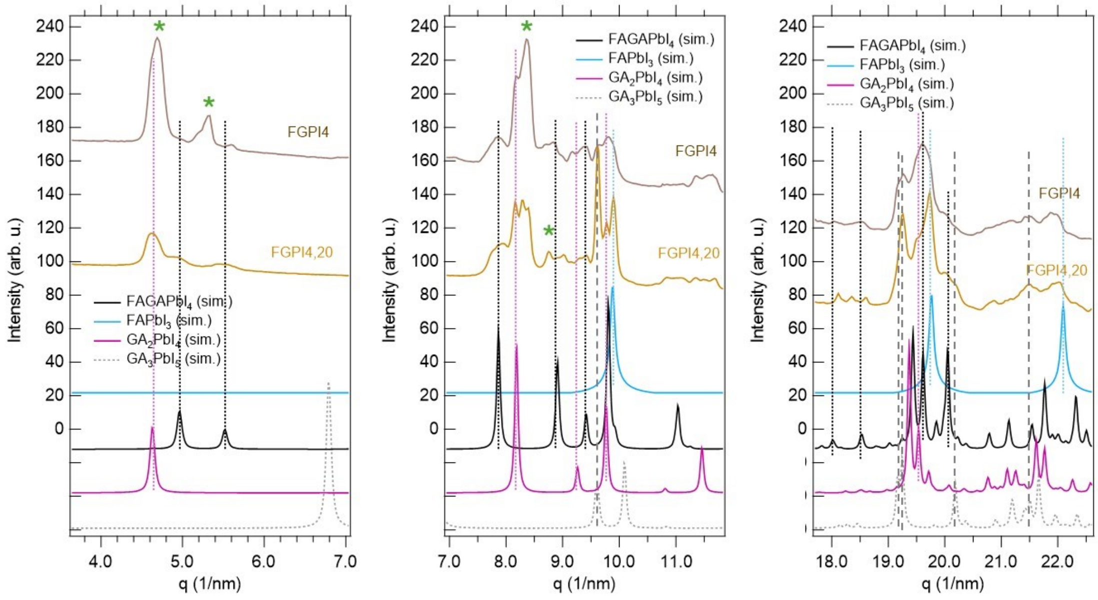

Fig. S5: Appearance of unknown peaks for FGPI-4,0 NWs marked with green stars across different q range**.**

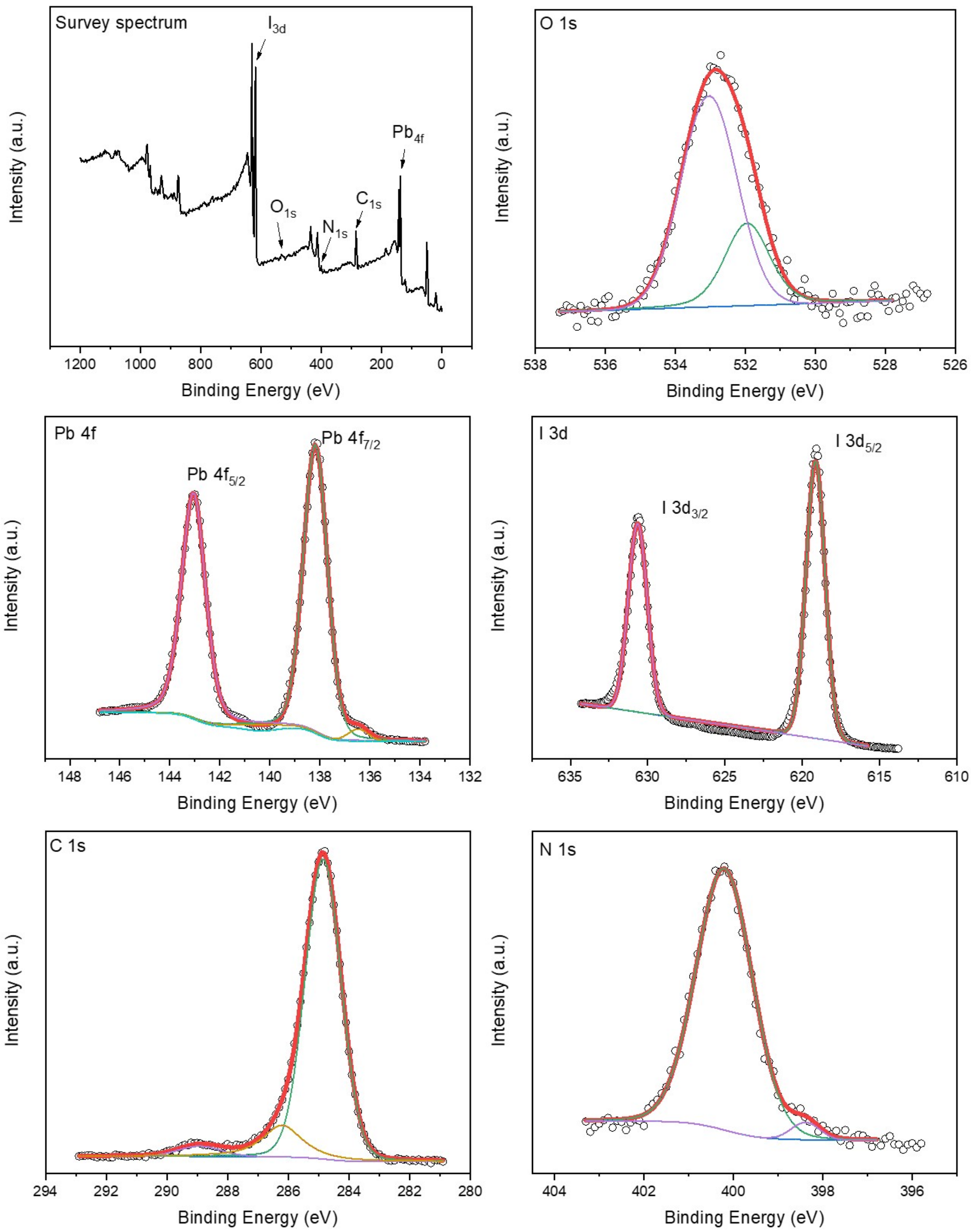

Figure S6. XPS measurements of FGPI NWs.

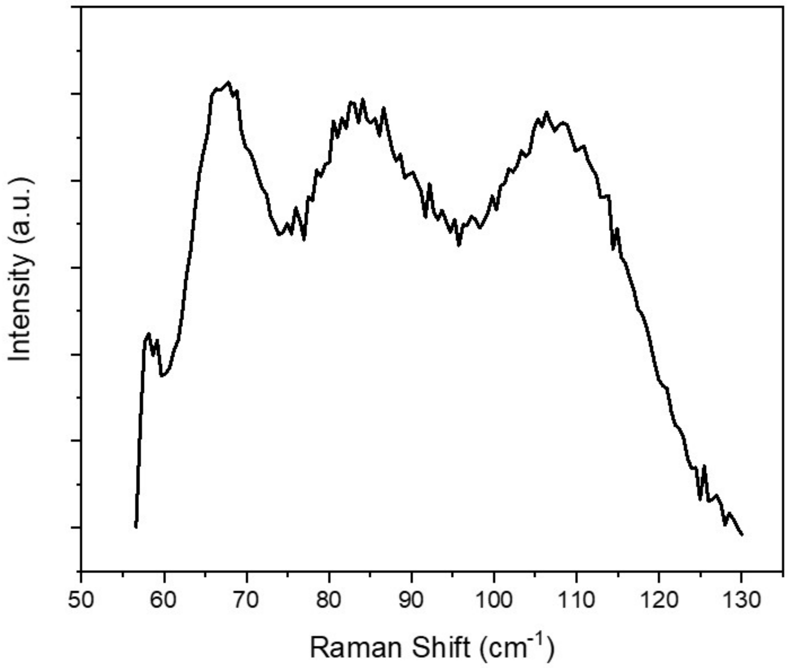

Figure S7. Raman spectrum of FGPI NWs

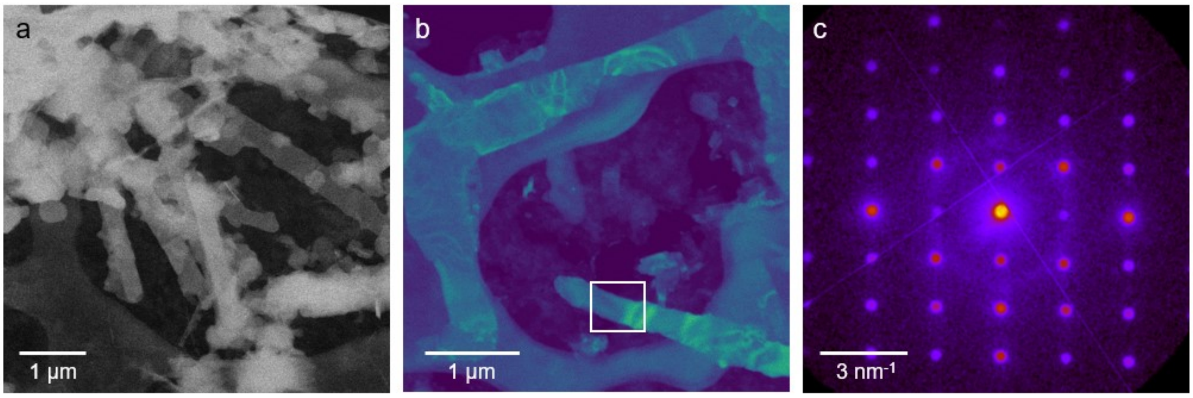

Figure S8. STEM imaging of the FGPI-4,0 sample. a) HAADF overview image depicting a nanowire morphology. b) Virtual ADF image reconstructed from a nano-beam 4D STEM dataset. c) Nano-beam 4D STEM diffraction pattern. The diffraction patterns were summed up from the 4D STEM dataset over the region indicated in the white rectangle in panel b. The diffraction pattern reveals that the crystal structure does not match with $FAGAPbI_4$ and indicates that the phase is $FAPbI_3$.

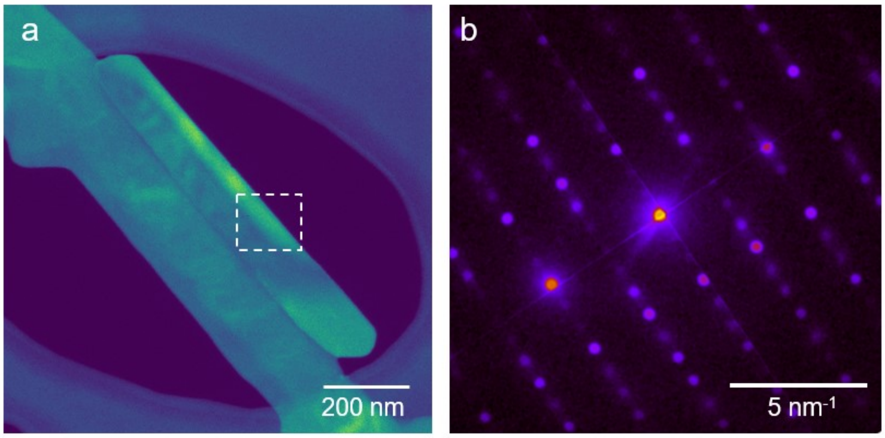

Fig. S9: STEM imaging of the FGPI-4,0 sample. a) Virtual ADF image reconstructed from a nano-beam 4D STEM dataset. b) Nano-beam 4D STEM diffraction pattern. The diffraction patterns were summed up from the 4D STEM dataset over the region indicated in the white rectangle in panel a. The diffraction pattern reveals that the crystal structure does not match with $FAGAPbI_4$ or $FAPbI_3$ but instead corresponds to an unknown phase, further highlighting the structural heterogeneity.

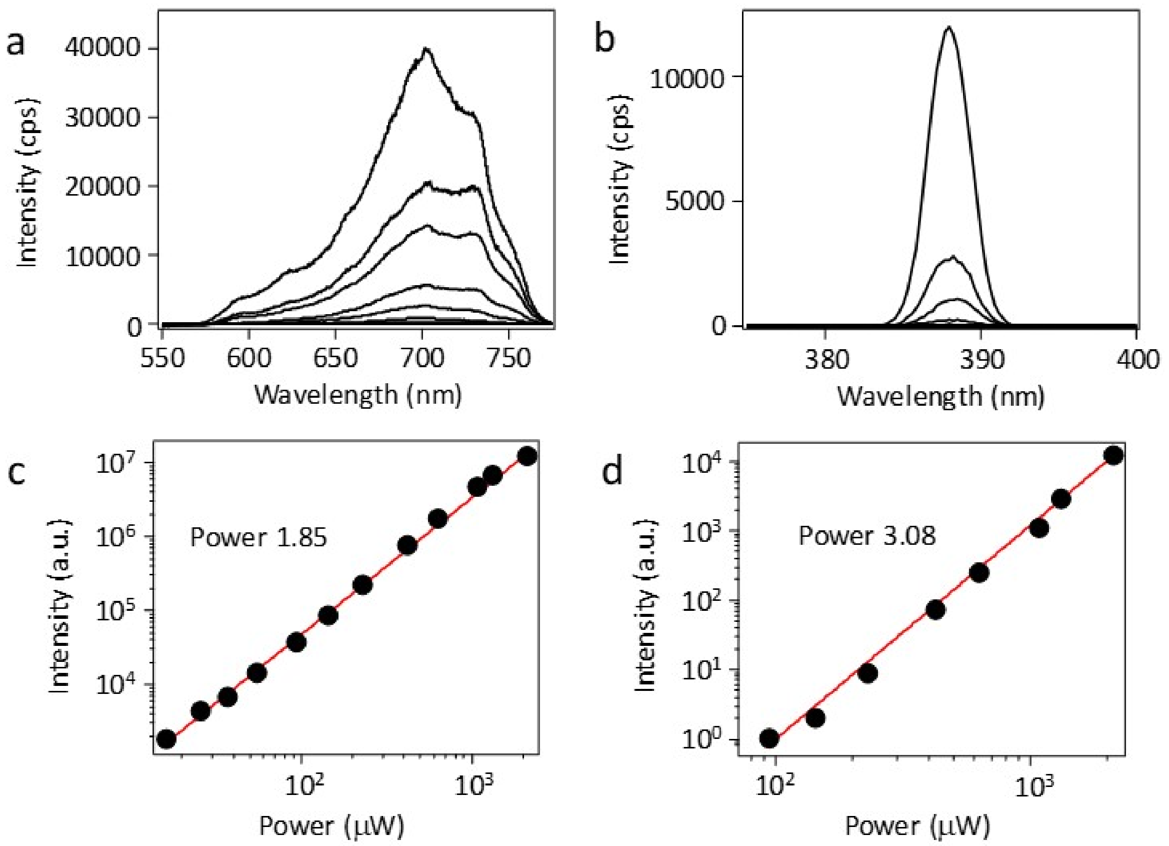

Figure S10. (a,b) TPPL (a) and THG (b) spectra under fs laser irradiation (1164 nm, 200 fs) with various power. (c,d) fs laser power dependence on TPPL (c) and THG intensities (d). TPPL intensity was determined by integrating TPPL spectra from 600 nm to 750 nm. THG intensity was determined by fitting THG spectra with a Gaussian function.

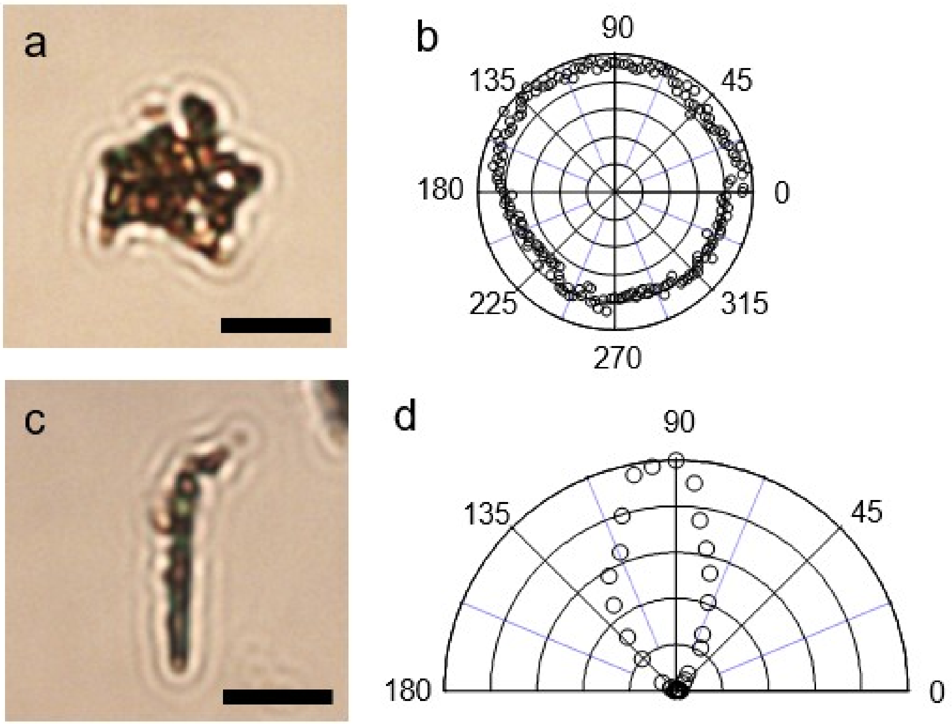

Figure S11. (a) Optical transmission image of a $FAGAPbI_4$ NW aggregate. Scale bar is 2 mm. (b) Polar plot of TPPL intensity of the NW aggregate under the light irradiation of linearized fs laser (820 nm, 120 fs, 100 mW). (c) Optical transmission image of a $FAGAPbI_4$ NW, which is vertically aligned. Scale bar is 2 mm. (d) Polar plot of THG intensity of the NW under light irradiation of a linearized fs laser (1164 nm, 200 fs, 100 mW).

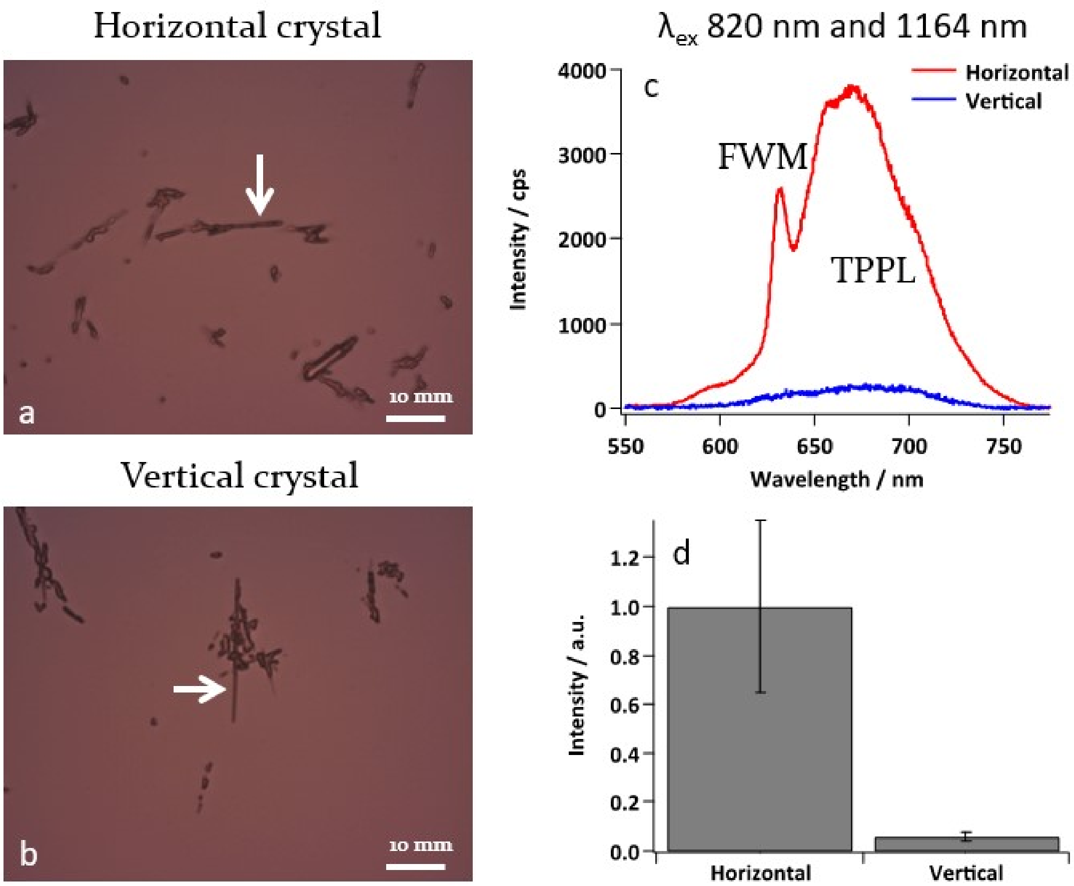

Figure S12 Optical transmission image of a $FAGAPbI_4$ NW in horizontal (a) and vertical (b) orientation. These were irradiated with a 820 nm and 1164 nm linearized fs laser. (c) Emission spectra of NWs with different orientations. The broad peak at 690 nm is due to two-photon photoluminescence (TPPL), and the sharp peak at 632 nm can be attributed to four-wave mixing (FWM). The FWM used here is called degenerate-four-wave mixing which means two of the three wavelengths going in are the same. This can be described as: $\omega = \omega_1 + \omega_1 - \omega_2$.[5] (d) Comparison of the relative intensity of both orientations.

References

1. Amenitsch, H. *et al.* First performance assessment of the small-angle X-ray scattering beamline at ELETTRA. *J. Synchrotron Radiat.* **5**, 506–508 (1998).
2. Jiang, Z. GIXSGUI: a MATLAB toolbox for grazing-incidence X-ray scattering data visualization and reduction, and indexing of buried three-dimensional periodic nanostructured films. *J. Appl. Crystallogr.* **48**, 917–926 (2015).
3. Burian, M., Meisenbichler, C., Naumenko, D. & Amenitsch, H. SAXSDOG: open software for real-time azimuthal integration of 2D scattering images. *J. Appl. Crystallogr.* **55**, 677–685 (2022).
4. Annys, A., Robert, H. L. L., Gholam, S., Hadermann, J. & Verbeeck, J. Removing constraints of 4D-STEM with a framework for event-driven acquisition and processing. *Ultramicroscopy* **277**, 114206 (2025).
5. Wolf, M. *et al.* Label-free visualization of heterogeneities and defects in metal–organic frameworks using nonlinear optics. *Chem. Commun.* **56**, 13331–13334 (2020).
